# Septal alcoholization in hypertrophic cardiomyopathy: about 11 cases

**DOI:** 10.11604/pamj.2017.27.196.9639

**Published:** 2017-07-13

**Authors:** Yibar Kambiré, Georges Rosario Christian Millogo, Claire Dauphin, Jean-René Lusson

**Affiliations:** 1 Department of Medicine and Specialties, National Hospital Blaise Compaoré, Ouagadougou, Burkina Faso; 2 University of Ouagadougou, Ouagadougou, Burkina Faso; 3 Cardiology Department, Teaching Hospital Yalgado Ouedraogo, Ouagadougou, Burkina Faso; 4 Department of cardiology and cardiovascular diseases, Teaching hospital of Clermont-Ferrand, France

**Keywords:** Hypertrophic obstructive cardiomyopathy, therapy, septal alcohol ablation

## Abstract

Outcomes of septal alcoholization in hypertrophic obstructive cardiomyopathy are not enough studied in all centers. The purpose of this study was to determine the outcomes of septal alcoholization in hypertrophic obstructive cardiomyopathy in our hospital. A retrospective and prospective descriptive study focused on all patients aged at least 18 years treated by alcohol septal ablation between July 2005 and June 2010 in the cardiology unit of Clermont-Ferrand teaching Hospital. The inclusion criteria were, hypertrophic obstructive cardiomyopathy with left ventricular outflow tract obstruction ≥ 50 mmHg, symptomatic despite optimal medical therapy. The clinical, paraclinical data and the results of alcohol ablation were collected from medical records of patients and a telephone conversation with the patients or their physicians. These data were analyzed by EPI info 6.04. Eleven patients with average age of 56.27 ± 15, 83 were included of which 81.8% of men. The main indications of alcohol septal were dyspnea stage NYHA II-IV (45.5%), lipothymia (18.2%) and invalidating angina (18.2%). Main electrocardiographic abnormalities were left ventricular hypertrophy and disorders of repolarization with 72.7% each. Minor conductive disorders were found in 45.5% of the cases. The left ventricular outflow tract obstruction was 98.18 ± 25.93 mmHg before alcohol septal ablation and 18.91 ± 31.97 mmHg after a follow-up of 25.64 ± 21.97 months. The success rate was 81.8%. Conductive disorders (45.5%) required the establishment of a definitive pacemaker in 36.4% of the patients. A cardiac defibrillator was implanted at 27.3%. Septal alcoholization was succesful.

## Introduction

The prevalence of Hypertrophic cardiomyopathy (HCM) is between 60 to 170/100.000 habitants [1, 2]. According to ultrasound studies it is familial in 55% of cases [3, 4]. A quarter (25-30%) of them is obstructive. The main clinical symptoms are diastolic heart failure, severe supraventricular or ventricular rhythm disorders and sudden death which burden the prognosis. This study reports the first five years monocentric experience of Clermont-Ferrand teaching Hospital on alcohol septal ablation. Its purpose is to assess the clinical and paraclinical outcomes of patients after alcohol septal ablation.

## Methods

It is a retrospective descriptive study covering the period of July 1^st^ 2005 to June 30^th^ 2010. It was conducted in the department of cardiology and vascular diseases of Clermont-Ferrand teaching Hospital.

## Patients

**Inclusion criteria**: Were consecutively included in the study all patients aged of at least 18 years presenting a hypertrophic obstructive cardiomyopathy (HOCM) with left intraventricular gradient greater than 50 mmHg at rest, remaining symptomatic after failure of optimal medical treatment and/or ventricular pacing, who have received an alcohol septal ablation during the study period.

**Non-inclusion criteria**: Patients with a contraindication to alcohol septal ablation and patients with non-obstructive or asymptomatic hypertrophic cardiomyopathy have been excluded.

The alcohol septal ablation procedure consists of several steps:

A temporary right ventricular stimulation catheter is set by the femoral vein route. Left heart catheterization confirms the dynamic left intraventricular obstruction and the extent of left intraventricular pressure gradient. Coronarography enables the selection of the artery to be alcoholized. The transthoracic intracoronary injection of contrast under echocardiographic monitoring guides the selection of this artery by showing the target area corresponding to myocardial obstructive bulge. The alcohol septal ablation is performed by a septal intracoronary injection of one milliliter (1ml) of ethanol 95° followed by a second injection of the same volume ten minutes later. The procedure is done under transthoracic echocardiographic (measurement of intraventricular gradient before and after alcoholiszation, choice of septal artery, looking for a pericardial effusion), hemodynamic and rhythmic monitoring. Monitoring in a cardiology intensive care unit with telemetry followed by a conventional hospitalization is carried out for at least four days. It focuses on the hemodynamic but importantly on the rhythmic looking rhythmic complications or conduction disorder. Post-septal alcoholization medical treatment is carried out with beta-blockers or calcium channel blockers. In case of supraventricular rhythm disorder, amiodarone is associated. The definitive pacemaker or cardiac defibrillator implantation is performed on a case by case basis according to the complications or associated risks. Post-septal alcoholization follow-up is done at one month then at six months for all patients. The follow-up is clinical, echocardiographic and rhythmic. The frequency of subsequent follow-up (annual or biannual) depends on clinical and paraclinical data, on risks and the presence or not of a pacemaker or cardiac defibrillator.

**Data collection**: It was conducted by searching in patient's medical and cardiac catheterization records and a telephone interview with the patient or a member of their families, their physicians and/or their cardiologists. The following variables were collected: age, sex, cardiovascular risk factors, cardiovascular history, comorbidities, clinical and paraclinical data (electrocardiographic, echocardiographic, hemodynamic for all patients). Holter monitoring, stress test and screening for genetic mutation have been performed for some patients. Indications, results of septal alcoholization as well as complications of the procedure and their treatment were also recorded.

**Definition**: The success of the procedure was defined by obtaining a left intraventricular gradient lower than 30 mmHg in immediate post-procedure or at medium term. Partial success was defined by a significant decrease in left intraventricular gradient without reaching the threshold of 30 mmHg.

**Data analysis**: Data were analyzed using EPI info 6.04 software. The results were expressed as mean ± standard deviation and relative frequency.

## Results

**Epidemiological data**: Eleven patients were involved in the present study. The mean age was 56.27 ± 15.83 years (extremes of 20 and 80 years). They were predominantly male (81.8%). The average time between the diagnosis of obstructive cardiomyopathy and septal alcoholization procedure was 51.72 ± 31.60 months (extremes of 1 and 97). Nine patients (81.8%) had at least one cardiovascular risk factor which were mainly age (54.5%), smoking (63.7%) and high blood pressure (27.3%). No family history of HOCM was found.

**Clinical data**: The main circumstances of HOCM discovery were the outcome of heart murmur (36.4%) and chest pain (27.3%) of which 18.2% were associated with a heart murmur and 9.1% with palpitations. Other circumstances of discovery were atrial fibrillation, heart failure and pre-therapeutic assessment for sismotherapy with 9.1% of cases each. Four complications (36.4%) were recorded before septal alcoholization. There were two cases of heart failure (18.2%), a recovered case of cardio-respiratory arrest (9.1%) and one case of atrial fibrillation (9.1%). Cardiac auscultation shown a systolic ejection murmur in all eleven patients at the time of septal alcoholization. The main clinical indications of septal alcoholization besides dynamic obstruction were exertional dyspnea class II-IV of New York Heart Association (NYHA) despite medical treatment (45.5%), malaise/pre-syncope (18.2%) and disabling angina with dyspnea (18.2%). Other indications were recovered cardio-respiratory arrest with persistence of intraventricular gradient under ventricular pacing (9.1%) and blood pressure intolerance to stress test (9.1%). Nine of the eleven patients were medicated with beta-blocker (81.8%) and two others with calcium antagonists (18.2%). Two of them had received pacemaker implantation for HOCM therapy before septal alcoholization.

**Paraclinical data**: The electrocardiogram was in sinus rhythm at the time of the procedure for all patients. It was abnormal for all of them. The main abnormalities were left ventricular hypertrophy and repolarisation disorders with 72.7% for each. Conduction disorders were found in 45.5% of cases. It was bi-fascicular block in 18.2% of cases, a left anterior hemiblock (9.1%), a complete atrioventricular block (AVB3) after his bundle ablation with ventricular stimulation in the context of HOCM and a paroxysmal AVB 3 appareled in a context of recovered sudden death. The transthoracic echocardiography has diagnosed HOCM with an intraventricular gradient exceeding 50 mmHg at rest for all eleven patients. This was an asymmetric HOCM in most cases with a mean septal thickness of 23.09 ± 4.76 mm (extremes of 16 to 33) against an average thickness of 15.91 ± 2.95 mm (12-22 mm) to posterior wall. It was a type 3 of Maron classification in 72.7% of cases. Types 1 and 2 accounted for 18.2% and 9.1% respectively. The average maximum left intraventricular gradient was 128.46 ± 32.50 mmHg (extremes of 87 and 193). Systolic function was preserved and even supernormal for all patients with mean ejection fraction of 74.82% ± 5.93 (extremes of 67 to 84%). The mitral flow was a disorder type relaxation (63.7%) and pseudo-normal type (27.3%). It was normal in 9.1% of cases. A systolic anterior motion of the mitral valve was found in 100% of patients with mitral regurgitation grade 1 to 3 in all eleven patients (100%). A mesosystolic closure of aortic valve was found in 54.5% of cases. The left atrium was dilated in 81.8% of patients while a slight to moderate pulmonary arterial hypertension was observed in 27.3% of them. A fluttering of anterior mitral valve's pillar was found in one case with a left intraventricular gradient of 193 mmHg.

A rhythm holter monitoring was performed for seven patients before septal alcoholization (63.6%). A burst of non-sustained ventricular tachycardia (VT) was recorded in one case and idioventricular rhythm in another case. Four patients had received at least one stress testing before septal ablation. It was pathological with blood pressure inadequacy to exercise in one case and not well tolerated with a stop at the first level in another case. Cardiac catheterization with intention to treat was performed in eleven patients. A septal artery suitable for target area was found in ten patients which is 90.9% of the cases. Significant coronary lesions were discovered in two patients (18.2%). Septal alcoholization has actually been performed in ten patients (90.9%). The results of the catheterization and septal alcoholization are summarized in table 1. Genetic mutation screening was performed in six patients and was positive for two patients. This was a DelE163 mutation of TNNT2 gene and a pGlu1218Glu mutation of exon 27 of the heterozygous gene MYH7. In both cases the genetic screening of the sister and the son (respectively for the first and second mutation) was negative. Search for familial recurrence was performed in seven patients. One patient had no first-degree relatives. There were no arguments to support family HCM based on clinical, electrocardiographic and echocardiographic data in six cases. A refusal was recorded among parents of another patient.

**Evolution**: The mean follow-up time of patients from the diagnosis of HOCM was 84.46 ± 44.15 months (range 6 to 143). The average follow-up duration after septal alcoholization was 25.64 ± 21.97 months (range 4 to 59). The average hospital stay length was 7.36 ± 4.39 days (range 4 to 18). Septal alcoholization was effective immediately or in the short term in 9 of 10 patients who actually received this therapy. Favorable clinical and hemodynamic course rate was 81.8% in all of eleven patients. One patient (9.1%) had clinical improvement. The last patient who didn't receive septal alcoholization because of lack of a suitable septal artery remained symptomatic (9.1%). No death was recorded during follow-up. Table 1 summarizes the clinical, paraclinical and therapeutic data before and after septal alcoholization. The mean peak of troponin after septal alcoholization was 3.6 ± 1.41 (range 2.31 to 5.81). The mean peak of creatine phosphokinase was 1194.82 ± 747.19 IU/l (range 430 to 2572). At the end of follow-up, six patients have developed at least one burst of ventricular tachycardia (54.5%) including four after alcoolization. Only one sustained VT treated with ATP was recorded. Among six patients who had at least one major risk factor for sudden death in addition to intraventricular gradient, three (27.3%) have received an implantable automatic defibrillator, of whom two for secondary prevention and one for primary prevention (for accumulation of three major risk factors for sudden death).

**Table 1:** Characteristics and outcomes of 11 patients with hypertrophic obstructive cardimyopathy before and after septal alcoolization

PeriodParameters	Prior-alcoolization	Immediate post-alcoolization	During hospital stay	Short term (1-3 months)	Medium term (4-8 months)	At end of follow-up
Maximum gradient (mmHg)	98.18 ±25.93 (52-130)	41.46±31.60	40.81±31.74 (11-94)	23,46±23.15 (0-63)	11,1±17.14	18.91±31.97 (2-109)
Maximum gradient < 30	0	5 (45.5%)	6 (54.5%)	8 (72.7%)	9 (81.8%)	9 (81.8%)
LVEF (%)*	74.81%±5.93 (67-84)	-	65,36±6.05% (53-74)	67.0±3.92	65.64	67.55±3.93 (60-75)
SAM**	11 (100%)	5 (45.5%)	5 (45.5%)	3 (27.3%)	2 (18.2%)	2 (18.2%)
Mitral valve regurgitation (1-3)	11 (100%)	3 (27.3%)	3 (27.3%)	3(27.3%)	3(27.3%)	3(27.3%)
Severe conduction disorder	2 (18.2%)	5 (45.5%)	4 (36.4%)	4(36.4%)	4(36.4%)	6(54.5%) ^‡^
VT***	2 (18.2%)	1 (9.1%)	0	0	2(18.2%)	6(54.5%) ^‡^
Other complications^†^	-	4 (36.4%)	-	1 (9.1%)		6(54.5%)
Pacemaker	2(18.2%)	-	4(36.4%)	-	-	6(54.5%) ^‡^
Implantable defibrillator	1(9.1%)	-	1(9 .1%)	-	1(9.1%)	3 (27.3%) ^‡^
Betablockers	9 (81.8%)	9(81.8%)	9(81.8%)	9(81.8%)	9(81.8%)	9(81.8%)
Verapamil	2(18.2%)	2(18.2%)	2(18.2%)	2(18.2%)	2(18.2%)	2(18.2%)
Clinical effectiveness	-	9(81.8%)	9(81.8%)	9(81.8%)	9(81.8%)	9(81.8%)

* LVEF = left ventricle ejection fraction

** SAM= systolic anterior motion

*** VT= ventricular tachycardia

† Other periprocedural complications ^†^: 1 pericarditis, 3 atrial fibrillation and infection of the arterial puncture site

‡ Rhythm/conduction disorders and pacemakers/defibrillators at end of follow-up including those present before and those that occured after alcoholization.

A pacemaker was implanted in four patients after septal alcoholization. The average time for permanent pacemaker implantation after alcoholization was 4.2 ± 2.56 days (range 2 to 8). Indications were AVB3 (two cases) and trifascicular block (two cases). The conduction disorders regressed in two patients within the same day of the procedure. The second case was after-hospitalization during the 5^th^ month monitoring of the previously implanted pacemaker. A transitional systolic dysfunction was observed in post procedure (ejection fraction of the left ventricle to 40%) in an elderly patient with multiple complications (AVB 3, paroxysmal atrial fibrillation, infection of the puncture site and minor pericardial effusion). Two clinical events justifying rehospitalization were recorded later after septal alcoholization: heart failure on bronchial infection in a patient with chronic obstructive pulmonary disease and recurrence of atrial fibrillation treated with amiodarone for the patient with septal alcoholization failure.

## Discussion

The treatment of hypertrophic cardiomyopathy is well codified today [5–8]. Septal alcoholization performed for the first time in 1994 by Sigwart and myectomie are the two most effective therapeutic means of reducing left intraventricular gradient in patients remaining symptomatic with left intraventricular obstruction despite an appropriate medical treatment. It is an alternative to myectomy for well selected patients without valvular insertion or pillar abnormalities. The selection of patients who are eligible for this treatment is performed by transthoracic echocardiography. This examination is also essential in the catheterization room for the choice of the septal artery and the identification of the target area. Finally, it is essential for post-septal alcoholization monitoring. Under these conditions septal alcoholization is feasible in 90% of patients with clinical efficacy and reduction of the left intraventricular gradient in the same proportions. In a literature review relating early experiences of the procedure success rate varies between 78 and 100% [9]. The periprocedural complication rate remained quite high and was dominated by conduction disorders ranging from complete AVB (0-70%) in complete right bundle branch block (11-58%) and complete left bundle branch block and/or anterior hemiblock (0 to 15%) [9]. However, the cardiac stimulation rate remains lower (8-38%) and hospital death rate low (0-4%) [9]. This mortality was mostly among elderly patients with several pathologies. In our study, among the eleven patients who underwent septal alcoholization, ten patients (90.9%) had a suitable septal artery and actually benefited from this therapy. Of the ten procedures performed the immediate effectiveness on left intraventricular gradient was 54.5%. In the end it was 81.8% of all candidate patients and 90% of those who have actually benefited from the procedure. These results are consistent with data from the literature reporting that the optimal outcome of septal alcoholization could be expected in several weeks or months, this allowing time for a sufficient ventricular remodeling [10].

A significant reduction of systolic anterior motion and mitral regurgitation was also obtained in 81.8% and 72.8% respectively. This evolution is typical. The rate of complications requiring permanent pacing has significantly improved (5-10%) along with technology and of the staff experience [8]. On the clinical level, nine of the ten patients who underwent alcoholization have shown a decrease of their symptoms with daily normal physical activity. Despite an hemodynamic failure the tenth patient experienced a decline in his dyspnea from stage III-IV to stage II of NYHA. This hemodynamic failure was observed in one patient where alcoholization was performed on the second septal artery, the first septal being unsuitable. The eleventh patient that haven't been not treated because of lack of suitable septal remained symptomatic at stage III of NYHA despite atenolol at 150 mg per day. Complications of septal alcoholization are dominated by atrioventricular or intraventricular conduction disorders [9–11]. They lead to the establishment of a permanent pacemaker in about 10% of cases [11]. In our series 54.5% of patients had AVB3 in periprocedural period. Final pacing was required in 36.4% of cases. This relatively high implantation rate could be partly explained by the fact that already 45.5% of patients had minor (27.3%) or severe (AVB3 = 18.2%) conduction disorder before the alcoholization procedure. The 18.2% AVB3 (including on removal of his) were already holders of pacemaker. The literature reports reducing rates of post-alcoholization definitive cardiac stimulation due to improving technology, progressive realization of procedure within five to ten minutes and the growing experience of the teams. Two of our five AVB3 (including an implanted) have regressed thereafter. Three periprocedural rhythmic complications (27.3%) were recorded in association with conduction disorders. It was an accelerated idioventricular rhythm (9.1%), and a paroxysmal atrial fibrillation (18.2%). Ventricular or supraventricular rhythm disorders are also reported in the literature [12].

We report bursts of ventricular tachycardia in three patients (27.3%) with recurrent ventricular tachycardia treated with ATP (9.1%) and two bursts of non-sustained new-onset ventricular tachycardia (18.2%). The VT even non-sustained is an independent factor of poor prognosis of HCM because it is responsible for sudden death [13]. Cases of fibrillation were recorded in our study. The relationship between these rhythm disorders and septal alcoholization is not well established, but it seems that the septal alcoholization does not cause more rhythm disorder due to limited myocardial scar compared to the coronary heart disease infarct and a decrease of QT after septal alcoholization [10, 11]. Apart from septal alcoholization, rhythm disorders are an integral part of HCM manifestations of which they are also a risk factor for sudden death or progression towards heart failure or strokes [7, 8, 14]. The other risk factors for sudden death are now codified by a risk score [15]. Out of our three cases of VT, recurrence has occurred in a patient with implantable defibrillator in the aftermath of a recovered sudden death before alcoholization. This patient had other associated VT risk factors: coronary heart diseases and chronic hypokalemia on secondary high blood pressure due to bilateral adrenal hyperplasia and stenosis of the renal artery. A case of heart failure occurred in a context of bronchial infection in a patient with chronic obstructive pulmonary disease. No death had occurred in our study. The risk of post- alcoholization death is generally low; less than 1% [11].

## Conclusion

The septal alcoholization was clinically and hemodynamically effective. One third of patients presented after a burst of non-sustained ventricular tachycardia and 27.3% received a new implantable cardioverter defibrillator. One third of patients received a new pacemaker for conduction disorder.

### What is known about this topic

- Septal alcoholization is indicated in symptomatic obstructive hypertrophic cardiomyopathy despite optimal medical treatment;
- The complication rate or permanent pacing varies according to the teams and their experience.

### What this study adds

- Our study takes stock of the first years of this technique in our center and confirms that prior conduction abnormalities increase the risk of permanent pacing.

## Competing interests

The authors declare no competing interest.
